# Martian magmatism from plume metasomatized mantle

**DOI:** 10.1038/s41467-018-07191-0

**Published:** 2018-11-15

**Authors:** James M. D. Day, Kimberly T. Tait, Arya Udry, Frédéric Moynier, Yang Liu, Clive R. Neal

**Affiliations:** 1 Scripps Institution of Oceanography, University of California San Diego, La Jolla, CA 92093 USA; 2 Department of Natural History, Royal Ontario Museum, Toronto, ON M5S 2C6 Canada; 3 Department of Geoscience, University of Nevada Las Vegas, Las Vegas, NV 89154 USA; 4 Institut de Physique du Globe de Paris, Université Sorbonne Paris Cité, Université Paris Diderot, 1 Rue Jussieu, 75328 Paris cedex 05, France; 5 Jet Propulsion Laboratory, California Institute of Technology, Pasadena, CA 91109 USA; 6 Department of Civil and Environmental Engineering and Earth Science, University of Notre Dame, Notre Dame, IN 46556 USA

## Abstract

Direct analysis of the composition of Mars is possible through delivery of meteorites to Earth. Martian meteorites include ∼165 to 2400 Ma shergottites, originating from depleted to enriched mantle sources, and ∼1340 Ma nakhlites and chassignites, formed by low degree partial melting of a depleted mantle source. To date, no unified model has been proposed to explain the petrogenesis of these distinct rock types, despite their importance for understanding the formation and evolution of Mars. Here we report a coherent geochemical dataset for shergottites, nakhlites and chassignites revealing fundamental differences in sources. Shergottites have lower Nb/Y at a given Zr/Y than nakhlites or chassignites, a relationship nearly identical to terrestrial Hawaiian main shield and rejuvenated volcanism. Nakhlite and chassignite compositions are consistent with melting of hydrated and metasomatized depleted mantle lithosphere, whereas shergottite melts originate from deep mantle sources. Generation of martian magmas can be explained by temporally distinct melting episodes within and below dynamically supported and variably metasomatized lithosphere, by long-lived, static mantle plumes.

## Abstract (web summary)

A unified model for the formation of martian rock types is required to understand Mars’s formation and evolution. Here the authors show that nakhlite and chassignite meteorites originate from melting of metasomatized depleted mantle lithosphere, whereas shergottite melts originate from deep plume sources.

## Introduction

Comparative planetology relies on assumptions that physical processes occurring for Earth can be used to compare with processes acting on other planetary bodies. This logic has been applied to Mars, where the broadly basaltic surface composition has been considered analogous to volcanism in terrestrial hotspot locations, including Hawaii^1^. Large volcanic edifices on Mars, including Olympus Mons, Alba Patera, Elysium, Arsia, Pavonis, and Ascraeus have been attributed to partial melting above mantle plumes^2,3^. Mars does not appear to have had plate tectonics, implicating long-lived and static volcanism above mantle plumes, in contrast to age-progressive volcanism occurring in oceanic and continental settings on Earth^4,5^. This difference, coupled with the distinct thickness and flexural rigidity of the elastic lithosphere on both planets, make assumptions of similar volcano-magmatic processes challenging. For example, observed pre-shield, shield, post-shield erosional, and rejuvenated stages of volcanism observed in Hawaii^6^ have not been recognized for Mars owing to the lack of plate motion.

Direct and precise geochemical observations for Mars are possible by analysis of meteorites recognized as having a martian heritage, from trapped Ar, Xe, and N_2_ signatures identical to the martian atmosphere^7–9^, and by their distinct oxygen isotope compositions^10^. These meteorites, which represent near-surface extrusive or hypabyssal intrusive rocks within the martian crust, including shergottites, nakhlites and chassignites, and ancient (4100–4400 Ma) crustal rocks (ALH 84001; NWA 7034/7533 and their pairs), are the ground truth for remote sensing^11,12^. Shergottites are the most geochemically diverse group of martian meteorites. They span a range of compositions, with evidence for long-term incompatible trace-element-depleted and trace-element-enriched reservoirs in Mars that were formed as early as ~4504 Ma^13,14^. Originally, the incompatible-element-depleted reservoir was considered to represent the mantle and, the incompatible-element-enriched reservoir, the crust^15^. This straightforward interpretation has been increasingly challenged by petrological, geochemical, and isotopic data, indicating the presence of both incompatible-element-depleted and incompatible-element-enriched martian mantle reservoirs^16–19^.

**Fig. 1:**
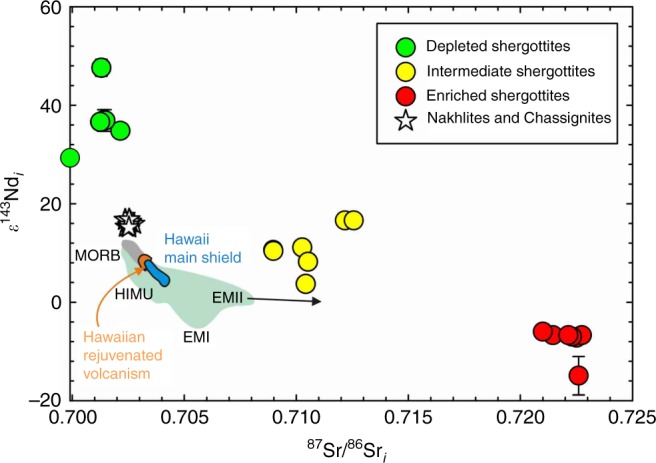
Comparison of the strontium and neodymium isotopic compositions of basaltic volcanic rocks at the time of their crystallization (*i* = initial crystallization time) from Mars and Earth. Terrestrial basalts include ocean island basalts (green field), mid-ocean ridge basalts (MORB; gray field), Hawaiian Main Shield (Mauna Loa, Mauna Kea; blue field), and Hawaiian Rejuvenated Volcanism (Honolulu volcanics, North Arch, Niihau, Kaula; orange field). Approximate terrestrial “HIMU” (high^238^U/^204^Pb), EMI and EMII (enriched mantle I and II) geochemical reservoirs are shown. ε^143^Nd_i_ = ([(^143^Nd/^144^Nd_i_)_sample_/(^143^Nd/^144^Nd_i_)_chondritic_]−1) × 10^4^. Data sources are given in the Methods section and error bars are smaller than symbols unless shown

The geochemical identity of shergottite source reservoirs is well expressed in ^87^Sr/^86^Sr–^143^Nd/^144^Nd space, where samples are corrected for ingrowth from ^87^Rb and ^147^Sm decay since crystallization (Fig. 1). Shergottites define long-term Rb and Nd-depleted, intermediate, and -enriched groups and, collectively, span compositions a factor of four greater than found in terrestrial ocean island or mid-ocean ridge basalts. The 1270–1420 Ma (average of 1340 ± 40 Ma) nakhlites and chassignites^20^ are isotopically distinct from shergottites, with depleted Sr and Nd isotope compositions. Nakhlites (clinopyroxene-rich mafic rocks) and chassignites (dunites) are petrogenetically associated low-degree partial melts from the same depleted mantle source^20–22^. The relationship of nakhlites and chassignites to shergottites is poorly understood. Both depleted shergottites and nakhlites have ^142^Nd enrichments consistent with early-formed Sm/Nd-enriched mantle sources^23,24^, but ^182^W and ^142^Nd data for nakhlites indicate a more complex origin than for shergottites that cannot be explained by early silicate differentiation alone^14^. Instead, late incompatible-element source enrichment must have occurred between major silicate differentiation at 4504 Ma and crystallization of the nakhlites at ~1340 Ma^14^.

In this study, we present a coherent geochemical dataset for 24 shergottites and 16 nakhlites that highlights fundamental differences in their incompatible trace-element compositions. These trace-element differences can be explained by melting of metasomatized mantle sources, likely the martian lithosphere, for nakhlites and chassignites, and deep mantle plume sources for shergottites. Combined with Sr-Nd isotope variations and geophysical evidence for high effective elastic thickness of the martian lithosphere (≥150 km), these data are consistent with generation of shergottites from mantle plume partial melts and nakhlites and chassignites from lower degree partial melting of hydrated mantle during loading of the martian lithosphere. These modes of formation for martian meteorites are analogous to Hawaiian main shield and rejuvenated volcanism, respectively. Instead of loading of moving oceanic lithosphere above a stationary hotspot, however, Mars manifests dynamic support of thick lithosphere by static mantle plumes, and the generation of large volcanic loads and the largest volcanoes in the Solar System.

## Results

### Compositions of shergottites and nakhlites

**Fig. 2:**
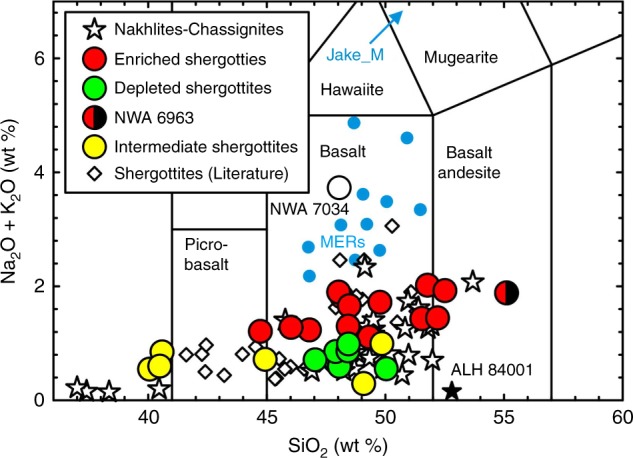
Total alkalis (Na_2_O + K_2_O) vs. silica diagram for martian meteorites and data for rocks from the Mars Exploration Rovers. Blue dots are Mars Exploration Rover data (MERs; ref. ^13^). Chassignite meteorites are star symbols with <41wt % SiO_2_. Data sources are this study and literature outlined in the Methods section. Analytical uncertainties are smaller than symbols

**Fig. 3:**
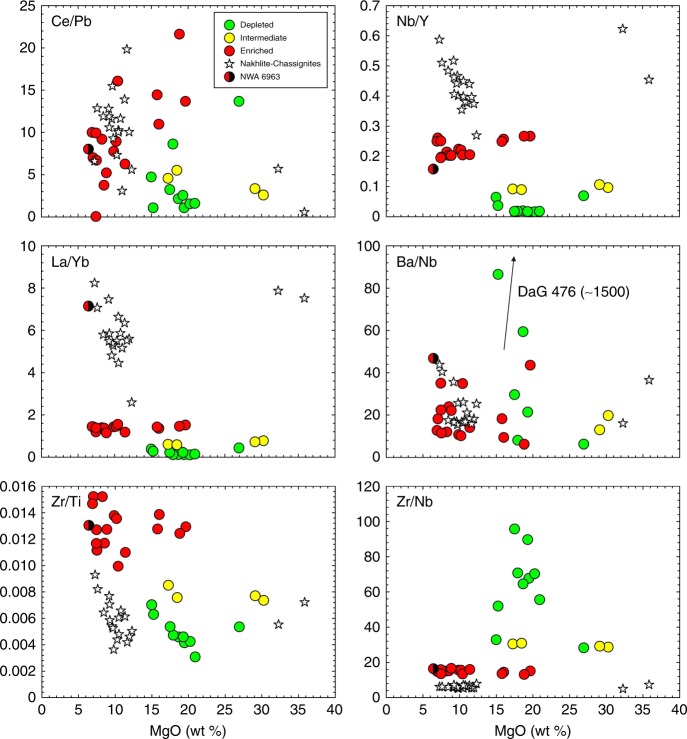
MgO vs. Ce/Pb, Nb/Y, La/Yb, Ba/Nb, Zr/Ti, and Zr/Nb for shergottites, nakhlites, and chassignites. Chassignite meteorites are star symbols with >30 wt % MgO. Data are from this study and analytical uncertainties are smaller than symbols

We present new data for 24 shergottite meteorites and compare these data with those obtained for 16 nakhlites and chassignites using identical analytical protocols that involved complete dissolution of samples (see Methods). Measurement under identical analytical conditions removes non-systematic error from analysis of individual meteorites in different laboratories. Shergottites range from picrobasalt to basaltic compositions, with enriched shergottites having elevated total alkalis (Na_2_O + K_2_O) relative to intermediate or depleted shergottites (Fig. 2). Nakhlites and chassignites span a wide range of compositions from low-SiO_2_ picrobasalt to basaltic andesite. Shergottites range from incompatible trace-element-depleted to trace-element-enriched compositions that are distinct from nakhlites and chassignites and are well expressed when comparing incompatible trace-element ratios (Ce/Pb, Nb/Y, La/Yb, Ba/Nb, Zr/Ti, and Zr/Nb) vs. indices of magmatic fractionation (MgO; Fig. 3).

**Fig. 4:**
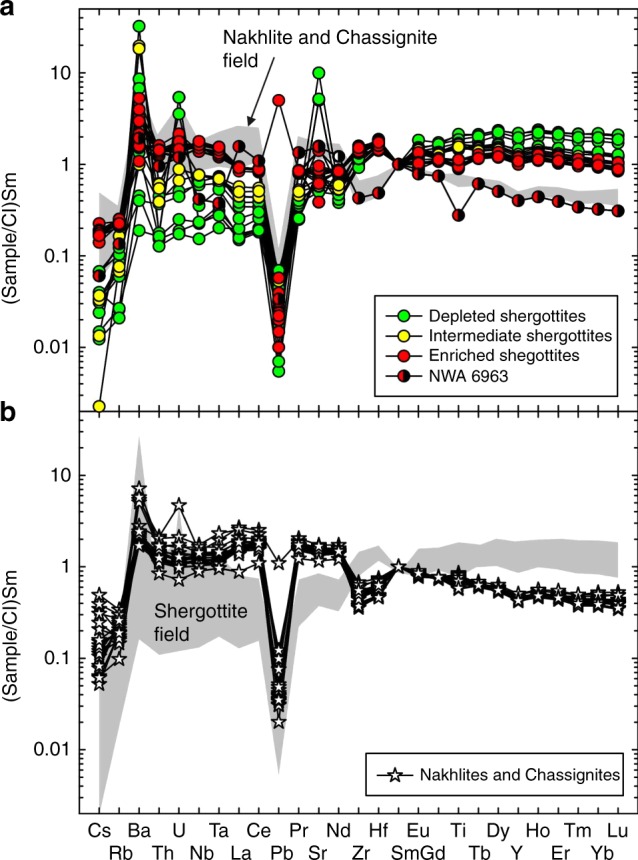
Double-normalized (to CI-chondrite and Sm) incompatible trace-element plots for martian shergottites, and nakhlites and chassignites. Shergottites are shown in **a** and nakhlites and chassignites are shown in **b**. Shergottite NWA 6963 is highlighted due to its similarity in incompatible element composition to nakhlites. Normalization to CI-chondrite from ref. ^39^. Analytical uncertainties are smaller than symbols

Depleted shergottites have lower absolute abundances of incompatible trace elements and rare earth elements (REEs) than intermediate or enriched shergottites and low CI-chondrite-normalized light REE/heavy REE (La/Yb_*n*_ = 0.114 ± 0.044; 1 SD), (Fig. 4). Ratios of La/Yb for intermediate shergottites (La/Yb_*n*_ = 0.39 ± 0.10) are lower than for enriched shergottites (La/Yb_*n*_ = 0.930 ± 0.078). In comparison, nakhlites and chassignites have a wider range of La/Yb_*n*_ = 0.99 ± 0.28, while NWA 6963 has La/Yb_n_ of 4.9. Hot desert meteorites have been variably affected by terrestrial alteration for Ba, Sr, and U, and NWA 2986 has a prominent Pb anomaly, which may also be due to terrestrial contamination. Sample NWA 6963 appears to be exceptional in that it has the texture and major-element geochemical composition of a differentiated shergottite (Fig. 2), but the incompatible trace-element composition of a nakhlite (Fig. 4).

### Distinct martian geochemical characteristics

Major-element and trace-element results for shergottites, nakhlites, and chassignites reinforce several previously identified characteristics of these rocks. Shergottite data highlights the incompatible-element-depleted, intermediate and -enriched compositions of samples^11^. Intermediate shergottites are the most mafic analyzed in this study, with enriched shergottites generally being more silica rich. Nakhlites and chassignites have light REE enrichment relative to the heavy REE^20^. The data also reveal fundamental differences in the geochemistry of shergottites vs. nakhlites and chassignites. Despite having similar Zr/Hf ratios (shergottites = 29.7 ± 4.5; nakhlites and chassignites = 30.6 ± 1.7), fractionation of Zr and Hf in shergottites is mirrored by nakhlites and chassignites. For example, calculated Zr* (Zr_*n*_/√[Nd_*n*_ × Sm_*n*_], where *n* is the normalized value to CI-chondrite) or Hf* (Hf_*n*_/√[Nd_*n*_ × Sm_*n*_]) are distinct between shergottites (Zr* = 1.6 ± 0.3; Hf* = 2.0 ± 0.4) and nakhlites and chassignites (Zr* = 0.4 ± 0.1; Hf* = 0.5 ± 0.1). Similar differences also exist for Nb (Nb* = Nb_*n*_/√[La_*n*_ × Th_*n*_]) and Ta (Ta* (Ta_*n*_/√[La_*n*_ × Th_*n*_]) between shergottites (Nb* = 1.4 ± 0.3; Ta* = 1.3 ± 0.3) vs. nakhlites and chassignites (Nb* = 0.8 ± 0.1; Ta* = 0.9 ± 0.1). As expected for melts from depleted mantle sources, nakhlite and chassignites have generally lower absolute abundances of Nb, Y, Zr, and Ta than intermediate or enriched shergottites, but higher relative abundances than the depleted shergottites.

## Discussion

The available samples for study from Mars are meteorites, many of which are finds and resided on Earth for significant periods of time (>100 years) prior to collection. The meteorites also have limited total mass making analysis of large (>30 g) well-homogenized aliquots, as is typically done for terrestrial igneous rocks, virtually impossible. Many of the martian meteorites that we studied show evidence for terrestrial alteration effects, including anomalously elevated large ion lithophile element (e.g., Cs, Ba, Sr) and U abundances. For example, the depleted shergottite DaG 476—a hot desert find—has Ba/Nb of ~1500, relative to Antarctic martian meteorite finds with Ba/Nb typically <30. Terrestrial alteration has had no effect on the majority of the REEs or high field strength elements (HFSEs) such as Nb, Ta, Hf, Zr, or Y. This is reflected in well-defined groupings for nakhlites and chassignites, and for incompatible element-enriched, intermediate, and -depleted shergottites for Nb/Y, La/Yb, and Zr/Nb (Fig. 3). For example, we find no systematic differences in the REE or HFSE inter-element ratios for the different geochemical types of shergottite, or between nakhlites and chassignites, from either hot or cold deserts.

The mode effect—where non-representative volumes of rock are chosen, resulting in greater variability in chemical measurements—is a significant issue if not recognized in meteorites owing to the small sample sizes typically studied by investigators (generally <5 g). To examine this effect, we analyzed separate aliquots of material from shergotittes NWA 7042, NWA 5298, NWA 7257, NWA 3171, DaG 476, and Tissint, and for nakhlites NWA 998, Nakhla, and MIL 090136. We find that while variations in absolute abundances of elements for separate aliquots can be large, up to 20% in some cases, the relative abundances and inter-element ratio variations are more limited (typically «10%). Occasionally, there are large variations in major-element abundances; for example, 50% variation for K_2_O in NWA 7042, and extreme heterogeneity between separate aliquots (e.g., NWA 998). Despite these variations, the inter-element ratios of key elements (e.g., Nb, Zr, Ta, Hf, Y) and the REE patterns of samples are unaffected, allowing assessment of the magmatic processes acting in Mars.

**Fig. 5:**
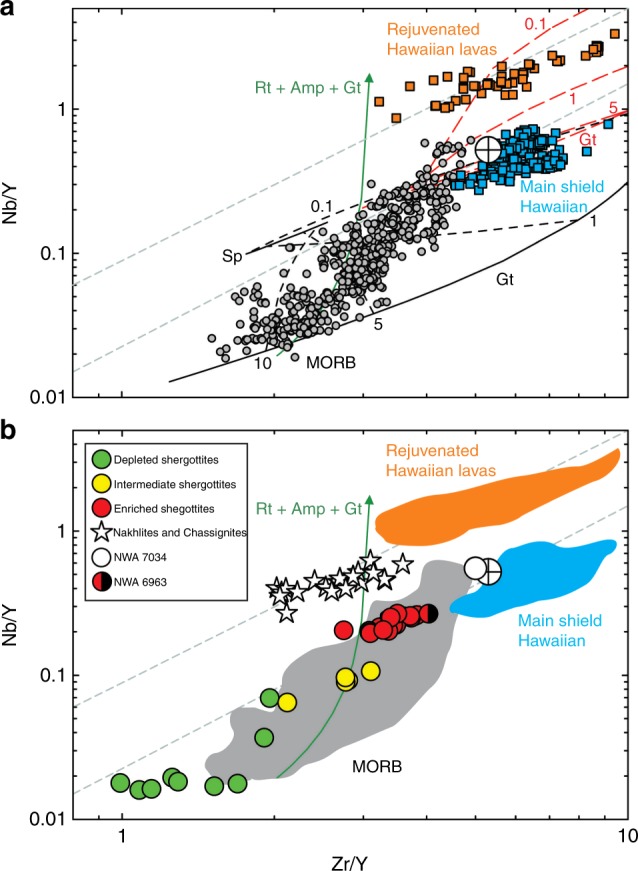
Partial melting processes in Mars and Earth. Niobium–zirconium–yttrium discrimination diagrams showing **a** terrestrial mid-ocean ridge basalts (MORB) and Hawaiian main shield building and rejuvenated phase lavas. Partial melt models show melting of a terrestrial depleted mantle source and a metasomatized mantle source, containing amphibole and rutile. Gray dashed lines denote envelope of total variation seen in Icelandic samples using this type of plot. Lines in red are a primitive mantle model, lines in black are a depleted mantle model, and the green line is the exhaustion vector for rutile (Rt), amphibole (Amp), and garnet (Gt) in a metasomatized martian mantle source. Numbers (0.1, 1, 5, 10) associated with dashed tie lines between pure garnet and spinel endmembers are partial melt increments in percent, and Sp is spinel. **b** Comparison of martian meteorites with terrestrial lavas. Crossed circle is the terrestrial continental crust composition. Model parameters and data sources are given in the Methods section and analytical uncertainties are smaller than symbols

A remarkable aspect of shergottite, nakhlite, and chassignite compositions is their similarity to geochemical differences observed between Hawaii main shield-stage and rejuvenated-stage volcanic rocks. Hawaiian shield-stage lavas form from the highest degrees of partial melting, above the plume conduit^25^. Rejuvenated-stage lavas occur after the main stages of volcanism and erupted peripheral to the Hawaiian plume center^26–30^. These rejuvenated alkalic basaltic lavas are incompatible-element-enriched low-degree partial melts, yet have Sr and Nd isotopic compositions requiring long-term depleted mantle sources relative to the shield source. These characteristics are like those for nakhlites and chassignites (Fig. 5). Such characteristics are difficult to explain by crustal contamination of parental magmas, since martian regolith (represented by NWA 7034/7533 and its pairs) is similar to terrestrial crust in having lower Nb/Y than nakhlites and chassignites. The variations in Nb/Y and Zr/Y for nakhlites are therefore common to the sense of fractionation for Hawaiian rejuvenated lavas and cannot be produced by crustal contamination.

A hallmark of Hawaiian rejuvenated-stage volcanism is that it begins after a volcanically quiescent period, requiring a mechanism to engender further melting. Several models have been proposed^28^, with the most popular invoking the depleted source as an intrinsic part of the Hawaiian plume that has been pervasively metasomatized by infinitesimally low-degree melts^28–30^. Maximum extents of rejuvenated melting occur during decompression of the mantle by lithospheric flexure, reaching a maximum ~200 km downstream from the plume^30^. For Mars, joint analysis of gravity and topography has been used to estimate effective elastic thickness of the lithosphere, typically interpreted as the depth of the 650 °C isotherm, or the point at which the material is too weak to support geological stresses over >10^8^ years. It has been shown that thickness of the martian lithosphere increases with age and decreases with radiogenic power from K, Th, and U decay^2^. In detail, the region with the most massive magmatism—Tharsis—appears to be dynamically supported, suggesting the presence of a mantle plume^2,3^, and consistent with relatively young (<100 Ma) volcanism in this region^31^. It has also been proposed that subsurface variations in load, including the presence of a depleted mantle composition, might exist underneath nearly all large martian volcanoes^3^.

We interpret shergottites to be analogous to shield-stage or pre-shield-stage and post-shield-stage, whereas nakhlites and chassignites are analogous to rejuvenated-stage igneous rocks. In the absence of plate tectonics, stationary plume-generated melting would be expected to strongly deplete portions of the martian lithosphere and mantle, as has been suggested from gravity and topography^3^. During plume impingement and maturation, eruption of large volumes of basaltic magmas would occur leading to loading of the lithosphere, a flexural bulge and a flexural moat around the volcanic load^32^. Flexural moats are observed around nearly all the Tharsis volcanoes from gravity data^33^ and are similar to, although significantly larger than, flexural bulges around Hawaiian-Emperor chain volcanoes^34^. To generate partial melts responsible for nakhlites and chassignites by lithospheric flexure, metasomatism of portions of depleted mantle would be required. Evidence for a water-bearing lithosphere is provided from its rheology^2^, and from addition of fluids into nakhlite and chassignites^20,22^.

Niobium/Y-Zr/Y systematics of nakhlites and chassignites support a model of partial melting of depleted mantle metasomatized by fluids. The relationships of high Nb/Y of Hawaiian rejuvenated and main shield lavas and nakhlites and chassignites and shergottites must reflect fundamental differences in Nb, Y, and Zr behavior during partial melting. Phases that have affinity for Nb, but less so for Zr or Y, include amphibole, rutile, ilmenite, and mica, of which mica and amphibole have been observed in nakhlite and chassignite melt inclusions^22^. Complete exhaustion of such phases during melting would explain the high Nb/Y of both nakhlite and chassignites and Hawaiian rejuvenated lavas. Models of 5–10% partial melting of primitive mantle reproduce Hawaiian shield compositions, whereas those of depleted mantle reproduce mid-ocean ridge basalt compositions (Fig. 5). Low-degree partial melts of a primitive mantle source could, in theory, generate Hawaiian rejuvenated lavas, but this is inconsistent with their depleted source character from Sr-Nd isotope systematics. The presence and complete exhaustion of limited quantities of hydrous phases (amphibole, mica) and rutile can generate high Nb/Y melts such as nakhlite and chassignites or Hawaiian rejuvenated volcanism.

The similarity between martian meteorites and terrestrial basaltic analogs implies important planetary comparisons. In detail, however, differences in isotopic compositions would suggest that the homogenization of mantle isotopic compositions by convective mixing has not been as effective for Mars as for Earth. The variation in Sr-Nd isotope systematics for martian meteorites, and the ~4500 Ma large-scale differentiation ages from these isotope systems^14,35^, indicate limited mantle stirring since planetary differentiation. This tectonic difference also explains the limited long-term time-integrated variation between Hawaiian main shield and rejuvenated lavas compared to the large difference in isotopic depletion for nakhlites and chassignites relative to intermediate or enriched shergottites.

Our interpretation of nakhlites and chassignites as forming during loading of the martian lithosphere provides predictive power. Martian rejuvenated volcanism should occur where lithosphere thickened and stabilized and where flexural uplift engendered partial melting. Sources of nakhlites and chassignites should be peripheral to plume volcanism at ~1300 to 1400 Ma and perhaps over longer periods of time for martian igneous rocks not currently sampled in the terrestrial meteorite collection. Additionally, shergottite crystallization ages (165–2400 Ma) generally preclude a direct link between nakhlites and chassignites. Nakhlites and chassignites come from a source that had prior melt depletion, and the previously extracted melts were likely similar to shergottites to explain mirrored elemental abundance patterns. Observations of 2400 to ~160 Ma shergottite magmatism on Mars supports continued and persistent plume magmatism for at least two billion years^13,36^. This link may be further strengthened by the observation that NWA 6963—a shergottite—has trace-element abundances like nakhlites, suggesting some geochemical affinities between magma types. We interpret the current suite of martian meteorites to represent at least two phases of plume-related magmatism. The distinctive nature of martian meteorites to remotely sensed martian surface samples, in particular the higher alkali contents of some Gusev and Gale crater samples^37,38^, possibly implies that alkali volcanism on Mars is driven by low-degree partial melting from metasomatism of martian lithosphere in response to mantle plume impingement, offering a mechanism for explaining the apparent differences observed between meteorites and mission data for Mars.

## Methods

### Sample preparation and analytical methods

New data were obtained for 24 shergottite meteorites, spanning the known range of incompatible element depletion and enrichment and showing a range of MgO contents, from 6.4 to 29 wt %. These data are compared with those obtained for 16 nakhlites and chassignites using identical protocols (Supplementary Data 1) and that were previously reported by Udry and Day^20^. For the desert finds, a Wells low-loss diamond wire saw was used to access material away from the fusion crust surface. For all materials, weathering or fusion crust surfaces were removed by crushing sample fragments with limited force, or by sawing, with sawn surfaces being thoroughly sanded with corundum paper, prior to generation of fine ground sample powders that were prepared using a clean alumina mortar and pestle.

Analytical procedures were undertaken at the Scripps Isotope Geochemistry Laboratory (SIGL). The standard technique used for all samples was digestion of 50 mg of sample powder in Teflon-distilled 27.5 M HF (4 mL) and 15.7 M HNO_3_ (1 mL) for >72 h on a hotplate at 150 °C, along with total procedural blanks and terrestrial basalt and andesite standards (BHVO-2, BCR-2, BIR-1a, AGV-2). The 50 mg powder aliquot was taken from a larger homogenized sample powder >0.5 g (up to 2 g) in all cases. Acid attack led to complete dissolution of rock samples, generating clear solutions, with no remaining solid material. Samples were sequentially dried and taken up in concentrated HNO_3_ to destroy fluorides, followed by doping with indium to monitor instrumental drift during analysis, and then diluted to a factor of 5000. Trace-element abundances were determined using a ThermoScientific iCAP Qc quadrupole inductively coupled plasma mass spectrometer (ICP-MS) and all data are blank-corrected. Long-term reproducibility of abundance data, based on the 3-year analytical campaign is better than 6% for most elements, except for Mo, Te, and Se (>10%).

Major-element abundances were determined on the same solutions used for trace-element abundance determination, using a 50,000× dilution factor. Major-element abundances were determined using a ThermoScientific iCAP Qc quadropole ICP-MS. For major elements, Si was derived by difference, with reproducibility of other elements measured on the BHVO-2 reference material being better than 3%, except Na_2_O (7.1%). Tait and Day^19^ measured ocean island basalt samples using both X-ray fluorescence and solution ICP-MS techniques, finding strong agreement between the two methods for MgO, Na_2_O, TiO_2_, Al_2_O_3_, and P_2_O_5_, and reasonable agreement for FeO, MnO, CaO, K_2_O, and SiO_2_. They also obtained precision typically better than 5% and accuracy from the accepted values of the BCR-2, BIR-1g and AGV-2 standard reference materials of better than 6%. The ICP-MS method is ideal for determination of major and trace elements in fresh samples with minimal alteration for which limited sample mass (<2 g) is available for study, such as meteorites. A caveat is that limited sample masses can lead to a mode effect, where non-representative volumes of rock are chosen, resulting in greater variability in chemical measurements, as described in the Discussion section.

### Complete sample dissolution

The procedures of digestion described above have been successfully and routinely used to fully dissolve terrestrial mafic igneous rocks^39^. For example, the method completely dissolves mafic igneous rock samples, like shergottites and nakhlites and chassignites. To confirm these results, we analyzed some of the standard reference materials and one martian meteorite (Nakhla) by first digesting the samples using a “Paar-Bomb digestion”. Identical protocols to the method described above were used, except that these materials were loaded into Teflon sleeves surrounded by a steel pressure vessel and heated to 180 °C in an oven for 72 h, and major-element abundances were not measured. As with the standard method of digestion, complete dissolution was accomplished. There are no observable differences, outside analytical uncertainties, or mode effect issues (e.g., replicate measurements of different fragments of Nakhla), for ratios of Zr/Y or Nb/Y, with the standard reference materials, BHVO-2 and BCR-2, reproducing within 2% for these ratios. Furthermore, a literature survey of martian shergottite, nakhlite, and chassignite meteorite trace-element abundance data show broad similarity with the results for the HFSE and REE presented here. The distinct Zr/Y, Nb/Y, Nb*, Hf*, Zr*, Ta*, and Nb* of nakhlites and chassignites vs. shergottites reflect true variations between the meteorites, rather than any analytical artifacts or terrestrial alteration effects.

### Data sources for Figs. 1, 5

Age-corrected Sr and Nd isotope data for shergottites, nakhlites, and chassignites in Fig. 1 were obtained from refs. ^13,35,36,40–50^ and references therein. Terrestrial ocean island basalt and mid-ocean ridge basalt Sr-Nd isotope data for Fig. 1 were obtained from refs. ^25–30,39,51–59^ and references therein. Since these data are from a range of laboratory sources, correction for non-systematic laboratory errors is not possible. Data for Fig. 5 are from refs. ^25–30,39,51–59^ and from ref. ^60^ for MORB and ref. ^61^ for the continental crust composition.

### Construction of the melt model

A partial melting model was constructed to evaluate the nature of the sources of rejuvenated lavas and nakhlites and chassignites vs. the sources of Hawaiian main shield-stage lavas and shergottites. Neither depleted (olivine (Ol), orthopyroxene (Opx), spinel (Sp), or garnet (Gt)) nor fertile mantle compositions (olivine, orthopyroxene, clinopyroxene (cpx), spinel, or garnet) can produce the trends in Fig. 5, using the partition coefficients summarized in https://earthref.org/KDD/ and in refs. ^62–64^ (Ol/Opx/Cpx/Gnt/Spinel/Amp/Phlog/Rutile/Zircon = [Nb] 0.0005/0.0013/0.0067/0.001/0.8/0.2/0.088/50/0; [Zr] 0.0031/0.013/0.11/0.14/0.6/0.2/0.17/4/100; [Y] 0.0057/0.074/0.4/3/0.05/1.4/0.018/0.76/0.7). Using a primitive mantle composition^65^ and a fertile mantle source assemblage (60% Ol, 17–20% Opx, 15% Cpx, 5–8% Gt or Sp) with modal melting proportions of phases can reproduce the Hawaiian shield-stage lavas between 3 and10% fractional partial melting. At low degrees of partial melting (~0.1%), such a source would also lead to a composition like Hawaiian rejuvenated lavas, but it would not be able to reproduce the Sr-Nd isotope compositions that required a long-term depleted mantle source.

We can reproduce the range of MORB compositions assuming a depleted MORB mantle composition^66^ and a correspondingly depleted mineral source composition (70% Ol, 25% Opx, 5% Gt or Sp) and dominant melting of olivine and orthopyroxene after exhaustion of garnet and spinel, with partial melts between 5 and 10% for the most depleted MORB samples. This trend can partly explain the martian shergottite compositions, although in detail, the depleted shergottites require an even more depleted source, evident from the highly unradiogenic Sr and radiogenic Nd compositions of these samples.

For shergottites, we can generate the range of Nb/Y and Zr/Y observed in the depleted, intermediate, and enriched shergottites by exhausting garnet (depleted shergottites) and retaining progressive amounts of garnet in the residue (intermediate and enriched shergottites), consistent with prior studies^14^.

Despite the ability of a primitive mantle composition partial melt model to generate melts similar to Hawaiian rejuvenated lavas, such a source is implausible given the unradiogenic Sr and radiogenic Nd isotope signatures of these lavas, suggesting a depleted source. To reproduce compositions similar to nakhlites and chassignites and rejuvenated Hawaiian lavas, a source with a mixture of phases that preferentially fractionate Zr—and in particular Nb—from Y are required. Phases with these characteristics include hydrous phases (amphibole, phlogopite) and rutile or ilmenite. Rutile has high affinity for Nb and, along with ilmenite, amphibole, and mica, have been recognized in terrestrial metasomatized lithosphere (MARID—mica–amphibole–rutile–ilmenite–diopside^67^). To date, there is no evidence for such occurrences in the martian mantle, but the presence of amphibole and micas in some martian meteorites suggests that hydrated mineral associations are possible in the martian mantle^22^. Modeled melting of a depleted mantle composition with ~1.5ppm Y and Nb/Zr = 0.01 with 0.05% rutile and 1% amphibole will result in rapid and complete exhaustion of exotic phases, and high resultant Nb/Y for a given Zr/Y value at between 0.1 and 1% partial melting of the martian-depleted mantle or the terrestrial-depleted mantle.

### High field strength elements and barium

The elements Ti, Zr, Y, and Nb are all high field strength elements (charge/radius ratio) that are not usually transported in aqueous fluids or strongly affected by metamorphic processes. These properties, and their systematic variation in lavas, make these elements important tracers of source variations in volcanic rocks^68,69^. In plots of these elements vs. MgO, there is clear distinction in the shergottite geochemical groups (enriched, intermediate, depleted) for La/Yb, Nb/Y, and Zr/Nb (Fig. 3). While Ba is similarly incompatible to Nb and La, it can be strongly modified by terrestrial alteration, most notably in depleted shergottites (e.g., DaG 476). The similarity in Ce/Pb could implicate melts with enriched shergottite-like characteristics as being similar to those that were lost from the source of nakhlites and chassignites.

### Crystallization ages

Crystallization ages for shergottites were taken from the literature (Supplementary Data 1) and Rb-Sr and Sm-Nd isotope crystallization ages were favored to plot the Sr-Nd isotope diagram (Fig. 1). Nakhlites and chassignites have been dated between 1270 and 1420 Ma, with an estimated mean age of 1340 ± 40 Ma on the basis of 40 individual age determinations (see ref. ^20^, and data in refs. ^40,41,45,47,49^^,^^70^).

## Electronic supplementary material

Supplementary Data 1

Peer Review File

Description of Additional Supplementary Data 1 Files

## Data Availability

The data that support the findings of this study are available within the paper and within the Supplementary Data file.

## References

[1] Zimbelman JR, Edgett KS (1992). The Tharsis Montes, Mars: comparison of volcanic and modified landforms. Proc. Lunar Planet. Sci. Conf..

[2] McKenzie D, Barnett D, Yuan DN (2002). The relationship between martian gravity and topography. Earth Planet. Sci. Lett..

[3] Belleguic V, Lognonne P, Wieczorek M (2005). Constraints on the martian lithosphere from gravity and topography data. J. Geophys. Res..

[4] Morgan WJ (1971). Convection plumes in the lower mantle. Nature.

[5] Coffin MF, Eldholm O (1994). Large igneous provinces: crustal structure, dimensions, and external consequences. Rev. Geophys..

[6] Clague DA, Sherrod DR (2014). Growth and degradation of Hawaiian volcanoes. U.S. Geol. Surv. Prof. Pap..

[7] Bogard DD, Johnson P (1983). Martian gases in an antarctic meteorite?. Science.

[8] Becker RH, Pepin RO (1985). The case for a martian origin of the shergottites: nitrogen and noble gases in EETA 79001. Earth Planet. Sci. Lett..

[9] Mahaffy PR (2013). Abundance and isotopic composition of gases in the Martian atmosphere from the Curiosity rover. Science.

[10] Franchi IA, Wright IP, Sexton AS, Pillinger CT (1999). The oxygen-isotopic composition of Earth and Mars. Meteorit. Planet. Sci..

[11] McSween H (2015). Petrology on Mars. Am. Mineral..

[12] Stolper EM (2013). The petrochemistry of Jake_M: a martian mugearite. Science.

[13] Lapen TJ (2017). Two billion years of magmatism recorded from a single Mars meteorite ejection site. Sci. Adv..

[14] Borg LE, Brennecka GA, Symes SJK (2016). Accretion timescale and impact history of Mars deduced from the isotopic systematics of martian meteorites. Geochim. Cosmochim. Acta.

[15] Jones JH (1989). Isotopic relationships among the shergottites, nakhlites, and chassigny. Proc. Lunar Planet. Sci. Conf..

[16] Blichert-Toft J, Gleason JD, Telouk P, Albarede F (1999). The Lu-Hf geochemistry of shergottites and the evolution of the martian crust-mantle system. Earth Planet. Sci. Lett..

[17] Symes SJK, Borg LE, Shearer CK, Irving AJ (2008). The age of the martian meteorite Northwest Africa 1195 and the differentiation history of the shergottites. Geochim. Cosmochim. Acta.

[18] Brandon AD (2012). Evolution of the martian mantle inferred from the Re-187-Os-187 isotope and highly siderophile element abundance systematics of shergottite meteorites. Geochim. Cosmochim. Acta.

[19] Tait KT, Day JMD (2018). Chondritic late accretion to Mars and the nature of shergottite reservoirs. Earth Planet. Sci. Lett..

[20] Udry A, Day JMD (2018). 1.34 billion-year-old magmatism on Mars evaluated from the co-genetic nakhlite and chassignite meteorites. Geochim. Cosmochim. Acta.

[21] Treiman AH (2005). The nakhlite meteorites: augite-rich igneous rocks from Mars. Chem. Erde.

[22] McCubbin FM (2013). A petrogenetic model for the comagmatic origin of chassignites and nakhlites: inference from chlorine-rich minerals, petrology, and geochemistry. Meteorit. Planet. Sci..

[23] Debaille V, Brandon AD, Yin QZ, Jacobsen B (2007). Coupled ^142^Nd-^143^Nd evidence for a protracted magma ocean in Mars. Nature.

[24] Caro G, Bourdon B, Halliday AN, Quitte G (2008). Super-chondritic Sm/Nd ratios in Mars, the Earth and the Moon. Nature.

[25] Bryce JG, DePaolo DJ, Lassiter JC (2005). Geochemical structure of the Hawaiian plume: Sr, Nd and Os isotopes in the 2.8 km HSDP-2 section of Mauna Kea volcano. Geochem. Geophys. Geosyst..

[26] Frey FA, Clague DA, Mahoney JJ, Sinton JM (2000). Volcanism at the edge of the Hawaiian plume: petrogenesis of submarine alkalic lavas from the North Arch Volcanic Field. J. Petrol..

[27] Bizimis M, Salters VJM, Garcia MO, Norman MD (2013). The composition and distribution of the rejuvenated component across the Hawaiian plume: Hf-Nd-Sr-Pb isotope systematics of Kaula lavas and pyroxenite xenoliths. Geochem. Geophys. Geosyst..

[28] Frey FA, Huang S, Blichert-Toft J, Regelous M, Boyet M (2005). Origin of depleted components in basalt related to the Hawaiian hotspot: evidence from isotopic and incompatible element ratios. Geochem. Geophys. Geosyst..

[29] Garcia MO, Weis D, Jicha BR, Ito G, Hanano D (2016). Petrology and geochronology of lavas from Ka’ula volcano: implications for rejuvenated volcanism of the Hawaiian mantle plume. Geochim. Cosmochim. Acta.

[30] Garcia MO (2010). Petrology, geochemistry and geochronology of Kaua’i lavas over 4.5 Myr: implications for the origin of rejuvenated volcanism and the evolution of the Hawaiian plume. J. Petrol..

[31] Hartmann WK, Neumann G (2001). Cratering chronology and the evolution of Mars. Sci. Rev..

[32] Watts, A. B. *Isostasy and Flexure of the Lithosphere* (Cambridge University Press, Cambridge, 2001).

[33] Genova A (2016). Seasonal and static gravity field of Mars from MGS, Mars Odyssey and MRO radio science. Icarus.

[34] Sandwell DT, Müller RD, Smith WHF, Garcia E, Francis R (2014). New global marine gravity model from CryoSat-2 and Jason-1 reveals buried tectonic structure. Science.

[35] Shih CY (1982). Chronology and petrogenesis of young achondrites, Shergotty, Zagami, and ALHA77005: late magmatism on a geologically active planet. Geochim. Cosmochim. Acta.

[36] Herd CDK (2017). The Northwest Africa 8159 meteorite: expanding the martian sample suite to the early Amazonian. Geochim. Cosmochim. Acta.

[37] Taylor, G. J., McLennan, S. M., McSween Jr, H. Y., Wyatt, M. B. & Lentz, R. C. in *The Martian Surface—Composition, Mineralogy, and Physical Properties* (ed. Bell, J. F.) 501–503 (Cambridge University Press, Cambridge, 2008).

[38] McSween, H. Y. et al. Alkaline volcanic rocks from the Columbia Hills, Gusev crater, Mars. *J. Geophys. Res.: Planets* **111**, 10.1029/2006JE002698 (2006).

[39] Day JMD, Pearson DG, Macpherson CG, Lowry D, Carracedo JC (2010). Evidence for distinct proportions of subducted oceanic crust and lithosphere in HIMU-type mantle beneath El Hierro and La Palma, Canary Islands. Geochim. Cosmochim. Acta.

[40] Papanastassiou DA, Wasserburg GJ (1974). Evidence for late formation and young metamorphism in the achondrite Nakhla. Geophys. Res. Lett..

[41] Nakamura N, Unruh DM, Tatsumoto M, Hutchison R (1992). Origin and evolution of the Nakhla meteorite inferred from the Sm-Nd and U-Pb systematics and REE, Ba, Sr, Rb and K abundances. Geochim. Cosmochim. Acta.

[42] Jagoutz E (1991). Chronology of SNC meteorites. Space Sci. Rev..

[43] Borg LE, Nyquist LE, Taylor LA, Wiesmann H, Shih CY (1997). Constraints on Martian differentiation processes from Rb-Sr and Sm-Nd isotopic analyses of the basaltic shergottite QUE 94201. Geochim. Cosmochim. Acta.

[44] Borg LE, Nyquist LE, Wiesmann H, Shih CY, Reese Y (2003). The age of Dar al Gani 476 and the differentiation history of the martian meteorites inferred from their radiogenic isotopic systematics. Geochim. Cosmochim. Acta.

[45] Shih CY, Nyquist LE, Wiesmann H (1999). Samarium-neodymium and rubidium-strontium systematics of nakhlite Governador Valadares. Meteorit. Planet. Sci..

[46] Shih CY, Nyquist LE, Wiesmann H, Reese Y, Misawa K (2005). Rb-Sr and Sm-Nd dating of olivine-phyric shergottite Yamato 980459: petrogenesis of depleted shergottites. Antarct. Meteortie Res..

[47] Nyquist LE (2009). Concordant Rb–Sr, Sm–Nd, and Ar–Ar ages for Northwest Africa 1460: a 346 Ma old basaltic shergottite related to “lherzolitic” shergottites. Geochim. Cosmochim. Acta.

[48] Morikawa N (2001). Rb-Sr isotopic systematics of lherzolitic shergottite Yamato-793605. Antarctic Meteorite. Antarct. Meteorite Res..

[49] Misawa K, Shih CY, Reese Y, Bogard DD, Nyquist LE (2006). Rb–Sr, Sm–Nd and Ar–Ar isotopic systematics of martian dunite chassigny. Earth Planet. Sci. Lett..

[50] Shafer JT (2010). Trace element systematics and 147 Sm–143 Nd and 176 Lu–176 Hf ages of Larkman Nunatak 06319: closed-system fractional crystallization of an enriched shergottite magma. Geochim. Cosmochim. Acta.

[51] White WM, Hofmann AW, Puchelt H (1987). Isotope geochemistry of Pacific mid-ocean ridge basalts. J. Geophys. Res..

[52] Nowell GM (1998). High precision Hf isotope measurements of MORB and OIB by thermal ionisation mass spectrometry: insights into the depleted mantle. Chem. Geol..

[53] Niu YL, Collerson KD, Batiza R, Wendt JI, Regelous M (1999). Origin of enriched-type mid-ocean ridge basalt at ridges far from mantle plumes: the East Pacific Rise at 11 degrees 20′N. J. Geophys. Res..

[54] Chauvel C, Blichert-Toft J (2001). A hafnium isotope and trace element perspective on melting of the depleted mantle. Earth Planet. Sci. Lett..

[55] Blichert-Toft, J., Weis, D., Maerschalk, C., Agranier, A., Albarède, F. Hawaiian hot spot dynamics as inferred from the Hf and Pb isotope evolution of Mauna Kea volcano. *Geochem. Geophys. Geosyst.* **4**, 8704 (2003).

[56] Abouchami W (2003). Lead isotopes reveal bilateral asymmetry and vertical continuity in the Hawaiian mantle plume. Nature.

[57] Weis D, Garcia MO, Rhodes JM, Jellinek M, Scoates JS (2011). Role of the deep mantle in generating the compositional asymmetry of the Hawaiian mantle plume. Nat. Geosci..

[58] Xu G, Frey FA, Clague DA, Weis D, Beeson MH (2005). East Molokai and other Kea-trend volcanoes: Magmatic processes and sources as they migrate away from the Hawaiian hot spot. Geochem. Geophys. Geosyst..

[59] Clague DA (2006). A submarine perspective of the Honolulu Volcanics, Oahu. J. Volcanol. Geotherm. Res..

[60] Gale A, Dalton CA, Langmuir CH, Su Y, Schilling JG (2013). The mean composition of ocean ridge basalts. Geochem. Geophys. Geosyst..

[61] Rudnick RL, Gao S (2003). Composition of continental crust. Treatise Geochem..

[62] LaTourrette T, Hervig RL, Holloway JR (1995). Trace element partitioning between amphibole, phlogopite, and basanite melt. Earth Planet. Sci. Lett..

[63] Blundy J, Dalton J (2000). Experimental comparison of trace element partitioning between clinopyroxene and melt in carbonate and silicate systems, and implications for mantle metasomatism. Contrib. Mineral. Petrol..

[64] Adam J, Green T (2006). Trace element partitioning between mica- and amphibole-bearing garnet lherzolite and hydrous basanitic melt: 1. Experimental results and the investigation of controls on partitioning behaviour. Contrib. Mineral. Petrol..

[65] McDonough WF, Sun SS (1995). The composition of the Earth. Chem. Geol..

[66] Workman RK, Hart SR (2005). Major and trace element composition of the depleted MORB mantle (DMM). Earth Planet. Sci. Lett..

[67] Dawson JB, Smith JV (1977). The MARID (mica–amphibole–rutile–ilmenite–diopside) suite of xenoliths in kimberlite. Geochim. Cosmochim. Acta.

[68] Pearce JA, Norry MJ (1979). Petrogenetic implications of Ti, Zr, Y and Nb variations in volcanic rocks. Contrib. Mineral. Petrol..

[69] Fitton JG (2007). The OIB paradox. Geol. Soc. Am. Spec. Pap..

[70] Cohen BE (2017). Taking the pulse of Mars via dating of a plume-fed volcano. Nat. Commun..

