# Supplementary material for: Martian magmatism from plume metasomatized mantle
Source: Nat Commun. 2018 Nov 15;9:4799. doi: 10.1038/s41467-018-07191-0 (PMC6237973; doi:10.1038/s41467-018-07191-0)
Supplement: Supplementary file 3 — Description of Additional Supplementary Data 1 Files [file 41467_2018_7191_MOESM3_ESM.docx]

Description of Additional Supplementary Files

Supplementary Data 1 - **Major- and trace-element abundance data for shergottites, nakhlites, chassignites and terrestrial rock standards**

Description: Major-, minor- and trace-element abundance data for shergottite, nakhlite and chassignite meteorites, and standard reference materials.
